# From “Silent Teachers” to Models

**DOI:** 10.1371/journal.pbio.1001971

**Published:** 2014-10-21

**Authors:** Roos Eisma, Tracey Wilkinson

**Affiliations:** Centre for Anatomy and Human Identification, University of Dundee, Dundee, Scotland, United Kingdom

## Abstract

For decades, embalmed cadavers have played an important role in teaching anatomy to the scientists and doctors of the future. Most anatomy departments use a traditional formaldehyde-based embalming method, but formalin embalming makes the bodies very rigid, which limits their usefulness for procedures other than dissection. A more recent embalming method developed by W. Thiel has allowed these “silent teachers” to take on a further role in applied anatomy research and teaching: to act as models for surgical training and medical research.

## Abstract 2

In this essay Roos Eisma and Tracey Wilkinson describe how the Thiel technique has expanded the range of applications in which embalmed human cadavers can be used.

## Introduction

In many parts of the world (Box 1), human anatomy is taught through whole body dissection, in which students have the opportunity to dissect organs and muscles, tracing their blood supply and innervation in all regions of the body. The bodies used for this purpose, generously bequeathed by donors to advance medical science and education, are often referred to as “silent teachers” [1],[2]. They teach the students what they cannot learn from models, textbooks, or 3-D programmes: variation between individuals, the effect of disease or lifestyle on the body, and the way different tissues feel and behave. Students also learn the legal framework around anatomy, including important lessons in ethics (how to treat human remains with dignity) and confidentiality (when not to discuss their activities openly) [3],[4]. For many of these young people, this is their first encounter with death, and although dissecting the body can be a daunting experience, it may also provide an opportunity for them to find out more about the donor's life. In most anatomical teaching facilities, a memorial event is held at the end of the year in which staff and students express their respect and gratitude to the donors and their families [5].

**Box 1. Body Donation throughout the World**

Donation practices vary considerably throughout the world, many being dependent on the cultural norms of the country. In the United Kingdom, body donation is strictly regulated, and every donor must have given written, fully informed, witnessed, and valid consent [41]. Countries in Europe vary considerably, with Germany, Austria, and the Netherlands requiring consent of the donor, while Italy has no real donation programme, and Portugal allows both bequests and the use of unclaimed bodies, as long as they have not signed an accord expressly forbidding organ donation [42],[43]. Serbian anatomical departments can accept when informed consent has been obtained either from the donor or the family and can also take unclaimed bodies [42]. In several countries in Africa, cadavers are sourced only through unclaimed bodies or criminals, while South Africa and Zimbabwe use a mix of donated and unclaimed bodies, and Libya imports unclaimed cadavers from India [44]. Body donation in the United States is governed at the individual state level, with procurement through informed consent. In many countries, public support strongly depends on the depiction of donation in the media [45].

Cadavers are employed not only in learning anatomy; they also serve an important role as an anatomical model in situations in which it is impractical, illegal, or unethical to work directly on patients. They are widely used for surgical speciality training [6],[7], with the advantage that they allow surgeons to make mistakes or try out new approaches. Cadavers contribute to the preclinical development of instruments and procedures used in surgery, allowing design teams to test new devices or techniques and identify areas of improvement in the early stages of development. Engineers and product designers have the opportunity to try their products in a hands-on manner, something that is not possible with an actual patient. While other models such as virtual reality, phantoms, or live animals are available, the most realistic model, from an anatomical point of view, is the human body itself.

To bridge the gap between death and use, it is necessary to preserve the bodies. The choice in preservation method greatly affects the future use of the cadaver. This paper describes the opportunities that a more recently developed method, Thiel embalming, can offer.

## Body Preservation Methods

Techniques for preserving the dead have been around for a long time, ranging from natural means, such as mummification or freezing, to artificial methods, such as immersion or arterial injection [8]. The focus in anatomical embalming, unlike that for funerals, is on long-lasting preservation of tissues rather than the maintenance of physical appearance. Many different combinations of chemicals have been used, with individual components acting, for example, as preservatives (to retain the structure of tissue), disinfectants (to halt decomposition), buffers, wetting agents, or dyes [8].

Table 1 gives the main advantages and disadvantages of commonly used preservation methods. Both embalmed cadavers and fresh-frozen tissue are used in education, training, and research. Embalmed bodies have the advantage of carrying minimal risk of infection and being suitable for prolonged use. Procedures are normally done on a complete body, providing a realistic experience. However, depending on the type of embalming, changes in mobility, colour, or tissue handling may occur [9],[10]. Fresh-frozen bodies, on the other hand, tend to be more realistic and flexible. Their disadvantages include the short period available before deterioration, the possibility of inadequate thawing, and, if separated body parts are used, the need for these to be clamped in place for the surgical procedure.

**Table 1:** Common body preservation methods and their key characteristics.

Preservation Method	Agents	Storage	Period of Use	Advantages	Disadvantages	Typical Use
Formalin	formaldehyde, with possible additions such as phenol and glycerine	room temperature	years	longevity, minimal infection risk, solid organs may be easier to handle	stiff, discoloured, unnatural texture, poor tissue plane preservation, odour, low grade carcinogen, not suitable for insufflation or ventilation	dissection based anatomy instruction and research
Thiel	glycol, various salts, boric acid, chlorocresol, formaldehyde (low levels), alcohol	room temperature after several months' immersion	years	flexible joints and tissues, realistic, minor tissue change, long lasting, ability to ventilate, preservation of colour	infrastructure required, time needed for embalming process, not all tissues lifelike	both short and long-lasting applications in teaching and research
Other soft embalming	various (e.g., glycerine, alcohol, formaldehyde, etc.)	fridge	months	somewhat flexible, some colour preservation	shorter lifespan, storage in fridge	
Fresh frozen	nothing	freezer	days	flexible, realistic, minimal tissue change	infection risk, need for full personal protective equipment, time needed for thawing, deterioration throughout usage period, mounting of body parts when not using full cadaver	short surgical training courses, short-term research

In general, stiff but durable formalin-embalmed cadavers are used for long-lasting dissection courses, while flexible but short-term fresh-frozen cadavers are used for surgical training. However, a soft-fix preservation method, developed by Thiel in the 1990s [11],[12], now produces cadavers that are both flexible and preserved long term and suitable for most current activities while additionally providing new opportunities. Thiel embalming is still relatively unknown and little used [13], but in recent years interest and uptake have increased.

**Figure 1:**
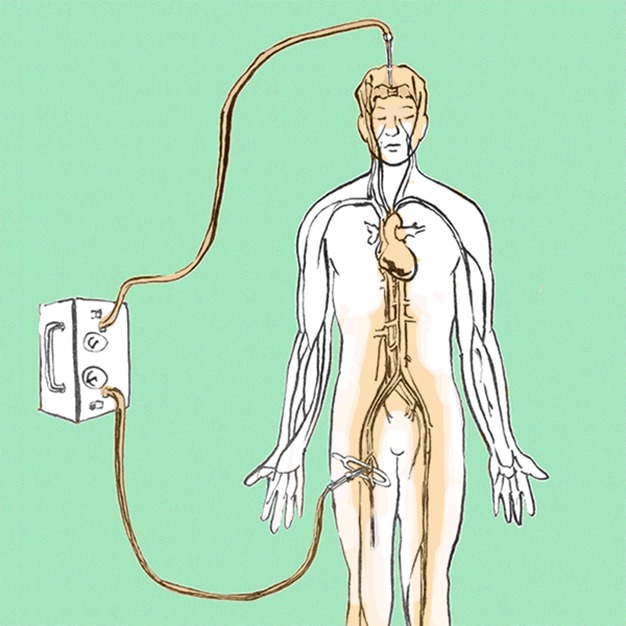
In the first step of the embalming process, the body is perfused with embalming fluids via the vascular system. Drawing by Emmanouil Kapazoglou.

Thiel embalming uses different fluids, which are all water-based mixtures with monopropylene glycol, ammonium nitrate, potassium nitrate, sodium sulphite, boric acid, chlorocresol, formalin, and, in the case of the perfusion fluids, also alcohol and morpholine [11],[12],[14]. The first step of the process is perfusion, which takes places shortly after arrival of the body in the anatomy department. Two fluids are infused simultaneously (Figure 1), one arterial (normally through the femoral or brachial artery) and one venous (normally through the superior sagittal sinus or brachial vein). After this, the bodies are submerged in a tank with embalming fluid for at least 4–6 months. They can then be stored in a sealed plastic bag until use, without need for refrigeration.

Thiel-embalmed cadavers are much more lifelike than formalin-embalmed cadavers. The flexibility of the joints is very good, and tissue planes and tissue colouring are retained well. Like formalin embalming, the cadavers are preserved long term and require minimal care while in use.

## Thiel-Embalmed Cadavers as an Anatomical Model in Teaching, Training, and Research and Development (R&D)

Because they are preserved long term, Thiel-embalmed cadavers are suitable for long-lasting dissection courses [14]. The softness and flexibility of the tissues provides a different experience compared to dissecting a formalin-embalmed cadaver, with in general a superior experience when studying the musculoskeletal system. The abdominal and thoracic organs are more realistic but considered by some to be more challenging [13],[14].

**Figure 2:**
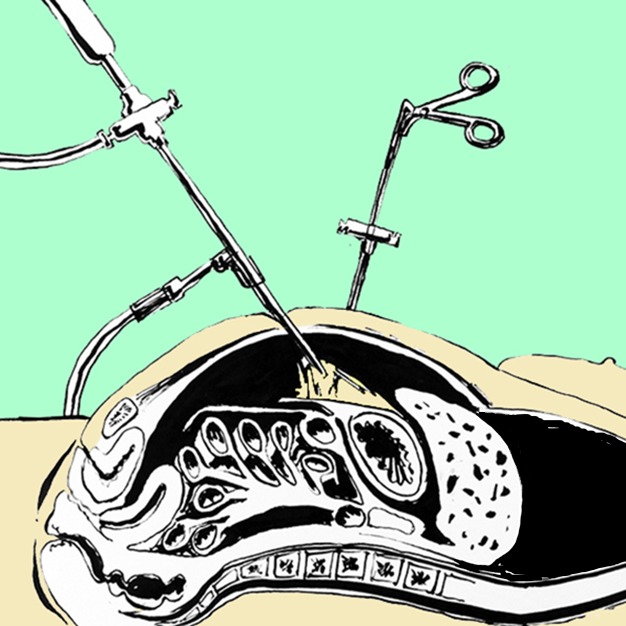
Thiel cadavers form a suitable model for laparoscopic procedures. In laparoscopy the abdomen is filled with gas; instruments, a light source, and a camera are then inserted through small incisions while the surgeon follows the procedure on a screen. It takes significant training to develop the skills and dexterity needed for this, and Thiel cadavers are regularly used to train surgeons. Drawing by Emmanouil Kapazoglou.

Thiel-embalmed cadavers have been shown to be suitable for a wide range of surgical procedures such as thyroid surgery [15], laparoscopy (Figure 2) [16]–[18], tendon repair [19], flap raising and microsurgery [20],[21], oral surgery [22], and orthopaedics, with more realistic properties than alternative soft embalming methods [9]. Limitations are the relatively poor preservation of the brain and the sometimes very soft texture of organs such as the uterus [14].

A major advantage of using Thiel embalming is the potential to make additional use of the cadavers for minimally damaging procedures that do not affect subsequent use by anatomy students or surgeons. For example, their flexibility makes them suitable for training in dental procedures [23], procedures such as intubation and ventilation [24], minimally invasive diagnostic procedures, ultrasound-guided anaesthesia [25],[26], suturing, biopsies, and various forms of endoscopy. While these procedures can be carried out on fresh-frozen cadavers, the costs involved (since unembalmed cadavers cannot be used multiple times) means that this is not always justified, especially for groups that would not normally have access to cadaver-based training, such as undergraduate students, paramedics, and other allied health professionals. The safe working conditions, with minimal exposure to infectious agents or harmful chemicals, mean that such user groups do not have to be trained in safety protocols or personal protective equipment.

Thiel-embalmed cadavers form an excellent model for development of products and techniques in any of the disciplines above, but their unique properties make them particularly suitable for applications in which both flexibility and durability are required. For example, the ability to reuse the same cadaver multiple times over a long period (and without need for defrosting or refreezing) allows more rigorous testing with direct comparison between products or operators [27],[28]. It also allows easy access for procedures that cause minimal damage to a cadaver, such as radiographic studies [29] or the development and evaluation of new techniques and products in ultrasound guided regional anaesthesia [26]–[28],[30]. Such studies can be carried out at short notice and with relatively low cost.

It can take a significant amount of time to set up a cadaver as a model for a specific procedure, and ideally this model should then be used for a longer period of time (Box 2). For example, once vascular flow has been established in part of the body, the cadaver can then be used repeatedly for training and development of interventional radiology procedures [31]—something that is not possible with either formalin-embalmed or fresh-frozen cadavers.

**Box 2. Thiel Cadavers as a Model for MR-Guided Focused Ultrasound Surgery**

At the Institute for Medical Science and Technology (IMSAT) in Dundee, researchers are developing cutting-edge medical treatments. One of the new techniques is MR-guided focused ultrasound surgery (MRgFUS) [46]. A high-power ultrasound beam is concentrated on, for example, a tumour, and the energy of the sound waves heats up the tissue and destroys the cells in the narrow focal point. Meanwhile, MR thermometry is used to direct and monitor the procedure.

To ensure that healthy tissue is not damaged, it is important that the beam is focused at the right location with an appropriate focal point size. However, when tumours in the liver are targeted, there are two problems: first, the patient is breathing during treatment so the diaphragm, and therefore the liver, goes through a cyclical motion; second, some or all of the liver is hidden behind the ribcage, which interferes with the beam.

Engineers are developing a range of techniques to address these problems, and they need a model to evaluate solutions while they go through iterations of refinement. Only once they can demonstrate that a technique is safe and likely to be effective can this go to clinical trials.

Thiel cadavers are the ideal model for this work. They are anatomically correct and can be ventilated to reproduce liver motion [24], blood flow can be simulated [31], they are suitable for MR imaging [39], and tissue heats up in response to MRgFUS [40]. Once the model has been set up, the cadavers can be used repeatedly over a longer period of time.

The ability to use a single cadaver for multiple consecutive procedures means their use can be more efficient, with a larger number of procedures being carried out on each individual cadaver, sometimes spanning a period of several years. This is in keeping with the moral obligation to make the best possible use of each precious donation and is particularly relevant in countries where access to donated bodies is relatively limited.

However, compared to fresh-frozen cadavers, Thiel-embalmed cadavers can display embalming-related changes, and it is important to understand these fully and know how they affect different uses of the cadaver—i.e., validation.

## Cadaver Model Validation

Validation relates to whether a model, product, or process meets the needs of its users, and in this context is the process by which an aspect of a cadaver (or a procedure carried out on it) is assessed and compared to a live patient situation. In the case of skin, for example, this may apply to its colour, to its resistance to puncture during suturing, or to the strength of the tissue when being manipulated by surgical instruments; in other words it depends on what is relevant to a specific use of the cadaver. In surgical training, it covers aspects such as “task fidelity” (how much the trained procedure resembles the real thing) [32] and “construct validity” (whether the model can discriminate between different levels of surgical experience) [33].

It is understood that no cadaver model can completely match a live patient in all aspects; for example, differences are likely in factors related to donor population (e.g., average age and health status) or caused by post-mortem and preservation processes. Physiological factors such as blood circulation, nerve conduction, or muscle contraction may be critical in certain studies, and these can be difficult or impossible to replicate. Since cadavers may be realistic in some aspects but not in others, care must be taken in determining the extent to which any training experience translates to the clinical equivalent. Product developers need to understand the limitations of the cadaver model to interpret the results of their experiments. Can these results be translated to clinical trials? Which observations can be attributed to embalming artefacts?

Validation is also closely linked to cadaver selection. Not all cadavers are identical; in the same way that people look different on the outside, they also differ on the inside, with potential variation in the course, route, and number of vessels or the size, shape, and position of organs, for example [34]. Further differences at the time of death relate to lifestyle, age, and health, such as the amount of fat stored in the body, the condition of vessels and muscles, and pathologies in organs and tissues. Individual cadavers can differ in how they respond to embalming, while variation in storage and use after embalming can introduce further disparity. As different procedures have different requirements, cadaver selection is an important tool in providing the most valid cadaver model.

Limited work so far has been done to validate Thiel-embalmed cadavers for different applications. Most studies evaluating surgical training on Thiel-embalmed cadavers are qualitative, relying on feedback from practitioners [16],[18],[26]. Some studies compare different cadaver models but do not directly compare these with live patients [15],[19],[25].

A number of small studies have demonstrated biomechanical changes to musculoskeletal structure [35]–[38] that indicate limitations to the use of Thiel-embalmed cadavers in biomechanical studies. A full understanding of changes to biomechanical properties of all tissues is needed to provide real insight into the strengths and limitations of the Thiel cadaver model and to underpin their use in product testing.

Validation is very specific to an application. For example, the case study described in Box 2 required (1) validation of the degree of liver motion through magnetic resonance (MR) imaging [24], which confirmed that the degree of motion of the liver is comparable to that of living individuals; (2) validation of MR imaging properties, which demonstrated that the high conductivity of Thiel embalming fluids affects some imaging sequences but not others [39]; and (3) validation of MR thermometry and tissue response to focused ultrasound surgery [40].

## Conclusions

Thiel-embalmed cadavers offer a unique combination of high fidelity (i.e., lifelike appearance, flexibility, and tissue quality and handling) and longevity. While they are not a universal replacement for either formalin-embalmed or fresh-frozen cadavers, there is much overlap with both of these in areas of use. Their real strength, however, is in applications in which both flexibility and durability are required, opening up new areas of research and training and extending this to groups other than anatomy students or surgeons.
